# Anti-N-Methyl-D-Aspartate Receptor (Anti-NMDAR) Encephalitis in Small Cell Lung Cancer: A Case Report

**DOI:** 10.7759/cureus.73713

**Published:** 2024-11-14

**Authors:** Hideya Itagaki, Momoka Hirano, Tomoyuki Endo

**Affiliations:** 1 Emergency and Disaster Medicine, Tohoku Medical and Pharmaceutical University Hospital, Sendai, JPN

**Keywords:** anti-nmdar encephalitis, autoimmune encephalitis, immunoglobulin, methylprednisolone, rituximab, small cell lung cancer

## Abstract

Anti-N-methyl-D-aspartate receptor (anti-NMDAR) encephalitis is an autoimmune encephalitis characterized by psychiatric and neurological symptoms. It often presents as a paraneoplastic manifestation and is rarely associated with small cell lung cancer. While treatment usually involves immunotherapy and treatment of underlying malignancy, the patient's condition can complicate treatment decisions. A 66-year-old man presented to the emergency department with a chief complaint of fever and impaired consciousness. Tests revealed anti-NMDAR encephalitis and small cell lung cancer. Despite immunotherapy, including steroids, intravenous immunoglobulin, and rituximab, chemotherapy was not possible due to the patient's poor condition. Immunotherapy treatment was continued, but there was no improvement in his state of consciousness, and death was confirmed on the 101st day of hospitalization. Anti-NMDAR encephalitis caused by small cell carcinoma is treated with immunotherapy and cancer therapy; only immunotherapy is not enough.

## Introduction

Anti-N-methyl-D-aspartate receptor (anti-NMDAR) encephalitis is an autoimmune encephalitis characterized by neuropsychiatric symptoms and autonomic dysfunction. Patients with anti-NMDAR encephalitis often present with tumor-associated symptoms, most associated with ovarian teratoma, but rarely reported in small cell lung cancer [1]. Treatment of anti-NMDAR encephalitis is immunotherapy, but tumor resection should be considered if the tumor is associated, as the resection of the tumor will speed improvement [2]. The mainstay of basic treatment for small cell lung cancer is chemotherapy and radiotherapy [3]. However, the treatment for small cell lung cancer with anti-NMDAR encephalitis remains undetermined. We report a case of anti-NMDAR encephalitis caused by small cell lung cancer treated with immunotherapy alone but without improvement in symptoms.

## Case presentation

A 66-year-old man was brought to the emergency room by his family with complaints of fever and impaired consciousness (abnormal behavior and disorientation). One week before his visit, he had insomnia and visited his local doctor, who prescribed several doses of zolpidem tartrate, a sleeping pill. Four days before his visit, he had flu-like symptoms and visited his local doctor again, where he was tested for coronavirus and influenza antigens, which were confirmed negative. Two days before his visit, he complained of tremors, elevated blood pressure, and fatigue and again visited his local doctor, who prescribed an antihypertensive drug: amlodipine besylate. The night before his visit, he suddenly got up and said he was going to work, which led to a call for emergency medical assistance. His medical history was hypertension only, and he was taking amlodipine besylate and zolpidem tartrate. His vitals on arrival were a Glasgow Coma Scale (GCS) of E4V3M5, a blood pressure of 172/95 mmHg, a pulse rate of 105 beats/min, a respiratory rate of 16 breaths/min, an oxygen saturation of 93%, and a body temperature of 37.9°C. Physical examination revealed no rigidity of the neck or involuntary movements, but the patient was prominent in sweating and repeatedly made unintelligible speech and behavior.

Based on the above, we suspected a central lesions such as encephalitis and meningitis and brain tumors and performed blood tests, imaging tests (CT/MRI), and cerebrospinal fluid (CSF) examination. Blood tests showed elevated leukocytes but normal or only slightly elevated C-reactive protein, procalcitonin, and interleukin-6, which are other inflammatory reactions. CSF examination showed elevated cell counts with monocyte predominance and elevated protein (Table 1).

**Table 1:** Blood and cerebrospinal fluid tests at admission ADA: adenosine deaminase; Alb: albumin; ALP: alkaline phosphatase; ALT: alanine aminotransferase; APTT: activated partial thromboplastin time; AST: aspartate aminotransferase; BS: blood sugar; BUN: blood urea nitrogen; Ca: calcium; Cl: chlorine; Cre: creatinine; CRP: C-reactive protein; Fib: fibrinogen; γGT: γ-glutamyl transpeptidase; Hb: hemoglobin; Hct: hematocrit; IL-6: interleukin-6; IgG: immunoglobulin G; K: potassium; LDH: lactate dehydrogenase; MCH: mean corpuscular hemoglobin; MCV: mean corpuscular volume; Na: sodium; PCT: procalcitonin; Plat: platelet; PT: prothrombin time; PT-INR: prothrombin time-international normalized ratio; RBC: red blood cell; T-bil: total bilirubin; TP: total protein; VitB1: vitamin B1; VitB12: vitamin B12; WBC: white blood cell

Parameter	Result	Reference value
Complete blood count data		
WBC	12.1	3.3-8.6 (×10^3^/μL)
RBC	4.57	4.35-5.55 (×10^6^/μL)
Hb	14.2	13.7-16.8 (g/dL)
Hct	42.4	40.7-50.1 (%)
Plat	296	158-348 (×10^3^/μL)
MCV	92.8	83.6-98.2 (fL)
MCH	31.2	30.5-34.2 (pg)
Fib	328	200-400 (mg/dL)
APTT	29.3	24-39 (sec)
PT-INR	1.06	0.90-1.15
D-dimer	0.66	0-1.0 (μg/mL)
Biochemistry data		
TP	7.9	6.6-8.1 (g/dL)
Alb	4.8	4.1-5.1 (g/dL)
T-bil	1.44	0.4-1.5 (mg/dL)
AST	26	13-30 (U/L)
ALT	28	10-42 (U/L)
ALP	66	38-113 (U/L)
γGT	67	13-64 (U/L)
LDH	193	124-222 (U/L)
BUN	33	8-20 (mg/dL)
Cre	1.19	0.65-1.07 (mg/dL)
Na	139	138-145 (mmol/L)
K	3.9	3.6-4.8 (mmol/L)
Cl	101	101-108 (mmol/L)
Ca	10.1	8.8-10.1 (mg/dL)
BS	188	70-110 (mg/dL)
VitB1	35	21-82 ng/mL
VitB12	252	197-771 pg/mL
Folic acid	8.4	4-20 (ng/mL)
CRP	0.07	0.0-0.14 (mg/dL)
PCT	0.08	0.0-0.05 (ng/mL)
IL-6	8.5	0.0-7.0 pg/mL
Cerebrospinal fluid test		
LDH	35	~25 (U/L)
Protein	96.6	~50 (mg/dL)
White cell count	238	0-3 (/μL)
Mononuclear	228	(/μL)
Multinuclear	10	(/μL)
IgG	10.1	1-4 (mg/dL)
IL-6	235.4	~2.4 (pg/mL)
ADA	4.9	~4 (U/L)
Herpes simplex DNA determination	<100	
Soluble interleukin-2 receptor	762	122-496 (U/mL)
Cryptococcus	(-)	
*Aspergillus* antigen	(-)	
Varicella-zoster virus DNA quantitation	<100	

**Figure 1:**
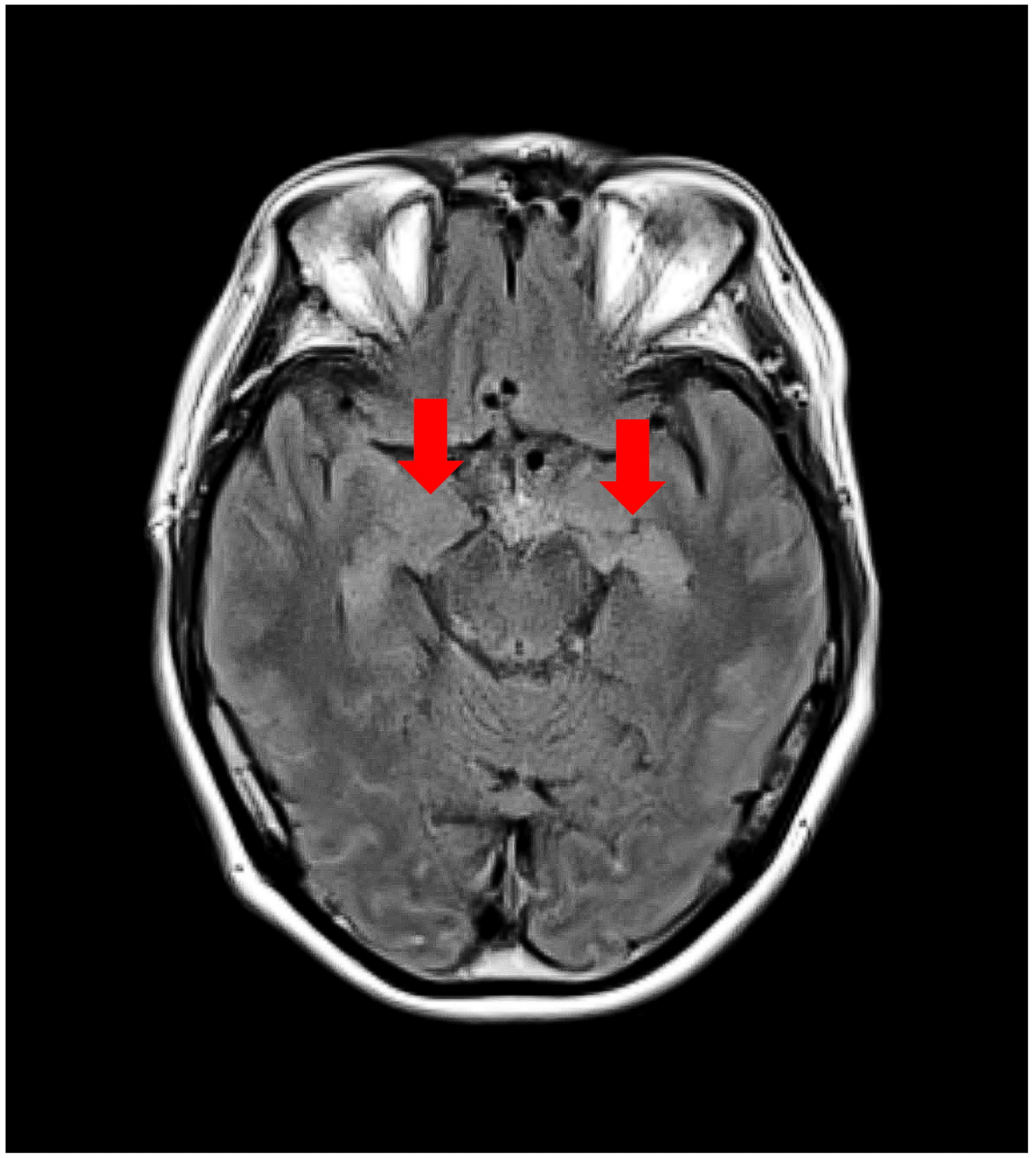
MRI (FLAIR) showed hyperintensities in both temporal lobes (red arrow) FLAIR: fluid-attenuated inversion recovery

Head CT showed no evidence of infarction or hemorrhage. Still, a head MRI fluid-attenuated inversion recovery (FLAIR) showed hyperintensities in both temporal lobes (Figure 1), and the patient was admitted to the hospital for close examination and the treatment of encephalitis.

**Figure 2:**
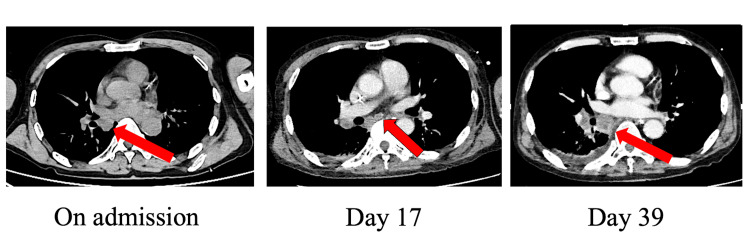
CT scan on admission and day 17 and day 39 of hospitalization: findings of lung tumor (red arrow) are gradually increasing in size

Intravenous methylprednisolone pulse (1000 mg for five days) and intravenous immunoglobulin (5 g for five days) were started on admission. On the third day of hospitalization, the patient was intubated and admitted to the ICU because of involuntary movements of the whole body and a worsening respiratory condition that led to mandibular breathing. An electroencephalogram (EEG) was performed, which showed diffuse slow waves of 1-2 Hz and no epileptic discharges. After intubation, the patient continued to have generalized body movements with involuntary movements controlled with sedatives midazolam and propofol and muscle relaxant rocuronium. On the same day, a spinal fluid examination at the hospital visit revealed positive anti-NMDAR antibodies, and the diagnosis of anti-NMDAR encephalitis was made. Later, a blood test revealed elevated markers of small cell carcinoma with neuron-specific enolase 25.1 ng/mL and progastrin-releasing peptide 116 pg/mL. A simple CT scan on the day of admission was unclear, so another CT scan was performed on the 17th day of admission. The results showed a mass shadow (2.5 cm) and adjacent lymphadenopathy on the dorsal surface of the right lung (Figure 2).

Small cell lung cancer was suspected, and sputum cytology was submitted, which confirmed the diagnosis of small cell lung cancer. On day 26 of hospitalization, the second course of steroid pulse and intravenous immunoglobulin was administered. The first dose of rituximab (500 mg per day) was started on day 31 of hospitalization. Thereafter, rituximab was administered three times, but there was no improvement in his state of consciousness. To improve the patient's consciousness, we discussed the treatment of small cell lung cancer with the Department of Pulmonary Medicine and Respiratory Surgery. However, the patient was deemed ineligible for chemotherapy, due to unconsciousness and ventilator dependence, and for radiation therapy, due to pleural dissemination, so only immunotherapy was continued. However, his impaired consciousness and involuntary movements remained unchanged, and the doses of midazolam/propofol and rocuronium could not be reduced. As the cancer grew, his condition worsened, and he died of circulatory failure on his 101st day in the hospital.

## Discussion

We learned the following from the experience of a patient with anti-NMDAR encephalitis caused by small cell lung cancer who died without neurological improvement after receiving only immunotherapy without chemotherapy or radiotherapy due to his general condition. First, anti-NMDAR encephalitis may occur as a first manifestation of small cell lung cancer. Second, immunotherapy alone did not improve neurological symptoms for anti-NMDAR encephalitis in small cell lung cancer.

Anti-NMDAR encephalitis is an autoimmune encephalitis caused by antibodies specific for the NR1/NR2 heteromers of the NMDAR, one of the glutamate receptors, and is generally more common in women, with a median age at onset of 21 years [4-6]. In younger women, it is often associated with ovarian teratoid species, while in those over 45 years of age, it is usually considered a carcinoma species rather than a teratoma [7]. About 40-70% of patients with anti-NMDAR encephalitis present with flu-like prodromal symptoms such as headache, fever, nausea, and vomiting, and within a few days to two weeks, they develop mental and behavioral symptoms, which are difficult to distinguish from psychiatric disorders, but, along with these symptoms, neurological findings: seizures, language dysfunction, involuntary movements, attention disorders, memory deficits, and autonomic neuropathy [5,8]. Although a definitive diagnosis of anti-NMDAR encephalitis requires the measurement of anti-NMDAR antibodies by CSF examination, three clinical diagnostic criteria were proposed by Graus et al. for the early initiation of treatment: The first is the rapid onset (<3 months) of at least four of the following six major symptom groups: mental, behavioral, or cognitive dysfunction; language dysfunction; seizures; abnormal movements, dyskinesia, or rigidity/postural abnormalities; decreased level of consciousness; and autonomic dysfunction or central hypoventilation. The second is at least one of the following tests: abnormal EEG (focal or generalized slow waves, disturbed fundamental waves, epileptic activity, extreme delta waves), increased CSF cells, or positive oligoclonal bands. Lastly, other diseases can be reasonably excluded [9]. This clinical diagnostic criterion can also be diagnosed in the presence of teratoma, and the criteria were met in the present case of small cell lung cancer [4]. Small cell lung cancer is a malignant tumor that accounts for about 13-15% of lung cancers with a high fatality rate due to rapid dissemination to the regional lymph nodes and distant organs [10]. Small cell lung cancer is said to be most frequently associated with paraneoplastic neurological syndrome (PNS), which includes limbic encephalitis, paraneoplastic encephalitis, and subacute sensory disturbance [10]. Encephalitis in small cell lung cancer is known to be associated with tumor antibodies such as anti-Hu and anti-CV2 antibodies and anti-gamma-aminobutyric acid-B (anti-GABAb) receptor antibodies, and an association with anti-NMDAR antibodies has also been suggested [11]. In the present case, psychiatric and autonomic symptoms, which are considered symptoms of anti-NMDAR encephalitis, appeared, but there were no symptoms clinically suspicious of small cell lung cancer. However, since 80% of PNS precede cancer detection by an average of 4-6 months, and since 50% of lung cancers are complicated by PNS and 80% of them are said to be small cell carcinoma, it may be necessary to differentiate lung cancer, especially small cell lung cancer, when the above mental symptoms and neurological symptoms appear [12,13].

Treatment of PNS in small cell lung cancer requires immunotherapy and cancer therapy (anticancer agents, platinum-etoposide, and radiation therapy) [13]. Table 2 shows the 12 cases of small cell lung cancer with anti-NMDAR encephalitis reported to date [6,11,14-20].

**Table 2:** Twelve cases of SCLC with anti-NMDAR encephalitis Me: memory loss; P/C: psychiatric behavior/cognitive dysfunction; Sp: speech dysfunction; Se: seizure; LoC: decreased level of consciousness; HV: hypoventilation; Aut: autonomic symptoms; Mov: movement disorder; CSF: cerebrospinal fluid; IVMP: intravenous methylprednisolone; IVIG: intravenous immunoglobulin; PE: plasmapheresis; RTX: rituximab; CTX: cyclophosphamide; Chemo: chemotherapy; Radio: radiotherapy; anti-NMDAR: anti-N-methyl-D-aspartate receptor; EEG: electroencephalogram; SCLC: small cell lung cancer

Author	Sex	Age	Prodrome	Symptoms								Laboratory study			Stage	Treatment for encephalitis					Treatment for SCLC		Outcome		Survival in months
				Me	P/C	Sp	Se	LoC	HV	Aut	Mov	CSF	EEG total with abnormal findings	MRI total with abnormal findings		IVMP	PE	IVIG	RTX	CTX	Chemo	Radio	Symptom improvement	Survival or not	
Uruha et al. [11]	M	68	Yes	No	Yes	No	No	Yes	Yes	Yes	Yes	Lymphocytic pleocytosis (29/mm^3^)	Yes	No	T3N2M0, stage IIIA	Yes	No	No	No	No	No	No	Yes	Death	6
Titulaer et al. [15]	M	76	No	Yes	Yes	Yes	No	Yes	Yes	Yes	No	-	-	-	-	No	No	No	No	No	No	No	-	Death	1
Boangher et al. [16]	F	66	No	Yes	Yes	No	No	Yes	No	No	No	Lymphocytic pleocytosis (18/mm^3^)	No	No	-	Yes	Yes	No	Yes	Yes	Yes	Yes	Yes	Survival	-
Jeraiby et al. [17]	F	62	No	No	Yes	Yes	No	Yes	Yes	No	Yes	Lymphocytic pleocytosis (20/mm^3^)	Yes	Yes	-	Yes	No	Yes	Yes	No	Yes	No	No	Death	2
Bost et al. [18]	F	67	-	Yes	Yes	Yes	No	Yes	No	Yes	Yes	Cells: 90/mm^3^	-	Yes	TXN3M0, stage IIIB	Yes	Yes	No	Yes	No	Yes	Yes	Yes	Death	36
	M	66	-	Yes	Yes	Yes	Yes	Yes	No	No	No	Cells: 21/mm^3^	-	Yes	T0N3M0, stage IIIB	Yes	Yes	Yes	No	No	Yes	No	No	Death	1
	F	62	-	Yes	Yes	No	Yes	Yes	No	Yes	Yes	Cells: 6/mm^3^	-	Yes	T2BN0M1, stage Ⅳ	Yes	No	Yes	Yes	No	Yes	No	No	Death	2
Sohal et al. [19]	M	72	Yes	Yes	No	No	No	No	No	No	No	Cells: 6/mm^3^: 93% lymphocytes	No	Yes	cT1cN2M0, stage IIIA	Yes	No	No	No	No	Yes	Yes	Yes	Survival	-
Kobayashi et al. [6]	M	61	No	No	No	No	Yes	Yes	No	No	Yes	Cells: 10/mm^3^	Yes	Yes	Stage Ⅱ~	Yes	No	Yes	No	No	No	No	No	Death	24
Akanuma et al. [20]	F	72	Yes	No	Yes	No	No	Yes	No	No	Yes	Cells: 25/mm^3^	No	Yes	T-N0M0	Yes	No	No	No	No	No	No	No	Death	1
Shobatake et al. [14]	F	63	No	No	Yes	Yes	No	Yes	No	Yes	Yes	-	-	Yes	cT2aN0M0, stage ⅠB	Yes	No	Yes	No	No	Yes	No	Yes	Survival	-
Our hospital	M	66	Yes	No	Yes	No	No	Yes	No	Yes	Yes	Cells: 238/mm^3^	Yes	Yes	cT1aN1M0, stage ⅡA	Yes	No	Yes	No	No	No	No	No	Death	3

Immunotherapy began with first-line treatments, including intravenous methylprednisolone, plasmapheresis, and intravenous immunoglobulin. Second-line treatments included rituximab and cyclophosphamide. For small cell lung cancer, chemotherapy and radiotherapy were also used. Immunotherapy was most frequently given as intravenous methylprednisolone (11/12 cases), intravenous immunoglobulin (6/12 cases), and plasmapheresis (3/12 cases), while the second-line treatments, rituximab (4/12 cases) and cyclophosphamide (1/12 cases), were less frequently given until the second-line treatment. Chemotherapy was used in 7/12 cases, radiotherapy was used in 3/12 cases, and both chemotherapy and radiotherapy were used in 3/12 cases. Four patients were treated with immunotherapy alone, and none were treated with cancer therapy alone. All survivors were treated with both immunotherapy and anticancer agents. Symptoms (neurological and psychological) improved in 5/12 patients, and 4/5 (80%) were treated with immunotherapy and anticancer agents. The same is true in other studies of encephalitis caused by small cell carcinoma. A study of limbic encephalitis caused by small cell carcinoma reported that even when immunotherapy (intravenous immunoglobulin and high-dose steroids) failed, complete or partial remission was achieved with anticancer therapy [13]. The same study also found that three out of four patients who received anticancer therapy without immunotherapy improved neurological symptoms [13]. Based on these findings, it can be inferred that anticancer drug therapy may be a key drug for anti-NMDAR encephalitis in small cell carcinoma. Considering the above, it is difficult to decide to use chemotherapy in cases such as the present case, in which the performance status has declined. Still, aggressive chemotherapy may improve the prognosis.

## Conclusions

Small cell lung cancer is often associated with tumor-associated syndromes, such as anti-NMDAR encephalitis, which may be the first manifestation of lung cancer. Treatment of anti-NMDAR encephalitis caused by small cell carcinoma also requires immunotherapy and chemotherapy, but immunotherapy alone may not improve symptoms. Since chemotherapy may improve symptoms, the decision to treat should be based on the patient's performance status at the time of treatment.
